# Use of the Hypoxia–Age–Shock Index at Triage to Predict Mortality in Geriatric STEMI Patients Undergoing Primary PCI

**DOI:** 10.3390/medicina62020365

**Published:** 2026-02-12

**Authors:** Man-Ju Ting, Wan-Ju Chao, San-Fang Chou, Shyh-Shyong Sim, Chih-Jung Chang, Chien-Chieh Hsieh

**Affiliations:** 1 Institute of Environmental and Occupational Health Sciences, National Taiwan University College of Public Health, Taipei 100, Taiwan; ruthting1@gmail.com; 2 Department of Cardiovascular Surgery, Cardiovascular Medical Center, Far Eastern Memorial Hospital, New Taipei City 220, Taiwan; 3 Department of Medical Research, Far Eastern Memorial Hospital, New Taipei City 220, Taiwan; 4 Department of Emergency Medicine, Far Eastern Memorial Hospital, New Taipei City 220, Taiwan; 5 Department of Biomedical Engineering, National Taiwan University, Taipei 100, Taiwan; 6 Department of Emergency Medicine, Ten Chan General Hospital, Chung-Li, Taoyuan City 320, Taiwan; 7 International Bachelor Program in Electrical and Communication Engineering, Yuan Ze University, Taoyuan City 320, Taiwan

**Keywords:** ST-segment elevation myocardial infarction, geriatrics, mortality, Hypoxia–Age–Shock Index, triage, percutaneous coronary intervention

## Abstract

*Background and Objectives*: Older adults with ST-segment elevation myocardial infarction (STEMI) experience disproportionately high mortality despite advances in reperfusion therapy. The Shock Index (SI) and Age–Shock Index (ASI) offer rapid hemodynamic assessment but do not address hypoxia. The Hypoxia–Age–Shock Index (HASI), which incorporates oxygen saturation (SpO_2_), may improve early mortality prediction in geriatric STEMI. *Materials and Methods*: This retrospective cohort study included adult STEMI patients receiving primary percutaneous coronary intervention (PCI) at a tertiary center from 2019 to 2023. A total of 711 patients were analyzed, including 254 aged ≥65 years. SI, ASI, and HASI were calculated using triage vital signs prior to intervention. The primary outcome was in-hospital mortality. Thirty-day mortality was analyzed as a pre-specified secondary endpoint using Kaplan–Meier survival analysis and multivariable Cox regression. Discrimination was assessed using ROC curves with pairwise AUC comparison by DeLong’s test. *Results*: Elderly patients showed higher creatinine and troponin T levels, lower hemoglobin, and elevated ASI and HASI values (all *p* < 0.001). They had increased rates of cardiogenic shock (26.8% vs. 14.0%), major adverse events (26.0% vs. 10.1%), and in-hospital mortality (9.4% vs. 3.7%, *p* = 0.003). Age ≥ 65 years independently predicted 30-day mortality (adjusted HR 2.59, 95% CI 1.34–5.04). Among indices, HASI demonstrated the highest discriminative performance (AUC 0.703 in elderly; 0.743 in younger patients). *Conclusions*: In geriatric STEMI, HASI demonstrated numerically higher discriminative performance for in-hospital mortality compared with SI and ASI, supporting its use as a simple and rapid triage tool.

## 1. Introduction

Despite substantial advances in reperfusion therapy and guideline-based care, patients presenting with ST-segment elevation myocardial infarction (STEMI) continue to experience significant short-term and long-term mortality, particularly in older populations [1,2,3]. Aging is associated with decreased cardiopulmonary reserve, increased comorbidities, and altered infarct pathophysiology, all of which complicate the acute management of STEMI and are associated with worse outcomes [4,5]. Identifying high-risk patients at the earliest possible point, such as at triage, remains a clinical imperative to guide timely intensive management and resource allocation [6,7].

Traditional risk stratification tools in STEMI, such as the GRACE score or TIMI risk index, often require multiple clinical, laboratory, and imaging variables and may not optimally capture the altered physiology of elderly patients in the emergency setting [8,9,10,11,12]. In contrast, simple bedside indices such as the Shock Index (SI) have demonstrated prognostic utility across acute cardiovascular conditions, including STEMI [13]. Admission SI has been associated with infarct size and adverse outcomes in these patients [14]. Building on this concept, the Age–Shock Index (ASI) has been shown to outperform SI in predicting in-hospital and six-month mortality in STEMI populations undergoing primary percutaneous coronary intervention (PCI). In a cohort of 983 patients, ASI demonstrated substantial discriminatory ability for in-hospital cardiac mortality (ROC AUC = 0.805), approaching the performance of the GRACE score [15].

However, age adjustment alone may still fail to capture the full spectrum of physiological derangement at presentation, particularly the impact of hypoxia, which reflects not only pulmonary involvement and impaired oxygen delivery, but also the downstream consequences of ventricular dysfunction, cardiogenic shock, and systemic hypoperfusion. Hypoxia on presentation has been independently associated with increased mortality in acute coronary syndromes, yet it has not been universally integrated into simple hemodynamic indices at the time of triage [16,17]. The concept of a Hypoxia–Age–Shock Index (HASI), which combines oxygen saturation (SpO_2_), age and hemodynamic parameters, therefore offers a novel and pragmatic approach to early risk stratification. To date, the application of HASI has been largely limited to non-cardiac populations (e.g., severe pneumonia or trauma) and has not been studied in geriatric STEMI cohorts undergoing primary PCI [18,19,20,21,22,23,24,25,26].

Given the disproportionately high mortality risk among older patients with STEMI and the need for rapid, bedside risk stratification tools in the emergency setting, we investigated whether the triage-derived HASI could improve mortality prediction in this high-risk population. The primary objective of this study was to evaluate the prognostic performance of HASI in comparison with the SI and ASI for predicting in-hospital mortality among STEMI patients aged ≥65 years undergoing primary PCI. A secondary objective was to characterize age-related differences in clinical presentation, hemodynamic profiles, and short-term outcomes, including 30-day all-cause mortality, between younger (<65 years) and elderly (≥65 years) STEMI patients, thereby contextualizing the clinical relevance of HASI in a geriatric cohort.

## 2. Methods

### 2.1. Study Design and Population

This retrospective cohort investigation was performed at a tertiary referral hospital and enrolled adult patients who presented with STEMI and received primary PCI between January 2019 and December 2023. Cases were consecutively identified from the institutional STEMI database and further confirmed through a detailed review of electronic health records. STEMI was diagnosed in accordance with the Fourth Universal Definition of Myocardial Infarction, requiring sustained ST-segment elevation of at least 1 mm in two or more contiguous electrocardiographic leads accompanied by elevated cardiac troponin concentrations [27].

Patients were excluded if they had undergone fibrinolytic therapy prior to interhospital transfer, had incomplete triage or laboratory information, or were missing initial measurements of oxygen saturation or blood pressure upon emergency department arrival. After applying these exclusion criteria, 711 patients remained eligible for analysis. Participants were categorized by age at presentation into non-elderly (<65 years; n = 457, 64.3%) and elderly (≥65 years; n = 254, 35.7%) groups. This age threshold was chosen to delineate the geriatric population and to facilitate evaluation of age-related differences in clinical characteristics and outcomes among STEMI patients treated with primary PCI.

### 2.2. Data Collection and Variables

Demographic data, clinical characteristics, vital signs, and laboratory results were retrospectively extracted from the institutional electronic health record system at the time of emergency department triage. Recorded clinical parameters included heart rate, systolic and diastolic blood pressures, respiratory rate, peripheral oxygen saturation (SpO_2_), body temperature, and Killip class at presentation. Laboratory variables included hemoglobin, platelet count, serum creatinine, cardiac troponin T, creatine phosphokinase (CPK), and creatine kinase–MB (CK-MB). Procedural variables such as door-to-balloon time, use of mechanical ventilation, and hospitalization duration were also recorded.

### 2.3. Definition of Physiologic Indices

Three hemodynamic indices were calculated at triage to quantify circulatory and oxygenation status. The SI was defined as the ratio of heart rate (beats per minute) to systolic blood pressure (mmHg). The ASI was calculated by multiplying the patient’s chronological age by the SI, thereby incorporating the effect of age into the assessment of hemodynamic instability. The HASI was computed by dividing the ASI by SpO_2_, integrating oxygenation into the age-adjusted shock model [6]. Notably, the SpO_2_ values used for HASI calculation were defined as the earliest available measurements obtained while patients were breathing room air, including those recorded in the prehospital setting by emergency medical technicians prior to the initiation of supplemental oxygen therapy for patients transported by ambulance. For patients arriving at the emergency department without prehospital oxygen supplementation, the first SpO_2_ measurement obtained at ED triage while breathing room air was used. Post-intervention SpO_2_ measurements obtained after oxygen administration were not used for analysis to avoid the confounding effects of supplemental oxygen therapy. All indices were derived from the first recorded triage vital signs prior to the administration of pharmacologic therapy or performance of PCI. This pragmatic marker at triage captures the initial physiological state and potential pulmonary congestion or systemic hypoperfusion associated with acute myocardial infarction.

### 2.4. Clinical Outcomes

The primary outcome of this study was all-cause in-hospital mortality, defined as death occurring during the index hospitalization, irrespective of length of stay. Secondary outcomes included 30-day all-cause mortality, requirement for endotracheal intubation, duration of mechanical ventilation, occurrence of cardiogenic shock, acute kidney injury (AKI), and length of hospital stay. Major adverse events (MAEs) were defined as a composite outcome including cardiogenic shock, in-hospital cardiac arrest, requirement for endotracheal intubation, acute kidney injury (according to KDIGO criteria), use of mechanical circulatory support (intra-aortic balloon pump or extracorporeal membrane oxygenation), or all-cause in-hospital mortality occurring during the index hospitalization.

Thirty-day mortality was defined as death from any cause within 30 days of the date of index hospital admission, regardless of discharge status. Patients who remained hospitalized beyond 30 days were considered to be continuously observed throughout the 30-day period, ensuring complete ascertainment of outcomes. For patients discharged before 30 days, post-discharge survival status within the 30-day window was determined through electronic medical record review and scheduled outpatient follow-up visits within our institution, which serves as the primary referral center for this patient population.

AKI was defined according to the Kidney Disease: Improving Global Outcomes (KDIGO) criteria, based on serum creatinine elevation and/or reduction in urine output [28].

### 2.5. Statistical Analysis

Continuous variables were expressed as mean ± standard deviation (SD) or median (interquartile range) depending on data distribution, and categorical variables were presented as numbers and percentages. Between-group differences were analyzed using Student’s *t*-test or Mann–Whitney U test for continuous variables, and the chi-square or Fisher’s exact test for categorical variables, as appropriate.

Survival curves for 30-day mortality, the secondary endpoint, were estimated using the Kaplan–Meier method, with differences between age groups evaluated via the log-rank test. Independent predictors of 30-day mortality were identified using multivariable logistic regression and Cox proportional hazards models, adjusting for clinically relevant covariates, including sex.

In addition, univariable and multivariable logistic regression analyses were performed to evaluate factors associated with in-hospital mortality. Univariable analyses were first conducted to screen candidate variables with potential prognostic relevance based on biological plausibility and prior literature, including HASI, age, sex, Killip class (or cardiogenic shock), serum creatinine, hemoglobin level, in-hospital cardiac arrest at presentation (IHCA), door-to-balloon time (D2B), left main coronary artery (LMCA) involvement, and multivessel coronary disease (defined as occlusion of ≥2 major coronary vessels).

A pre-specified multivariable logistic regression model was then constructed to assess the independent association between HASI and in-hospital mortality. Covariates were selected a priori based on clinical relevance, prior evidence, and statistical significance in univariable analyses. To minimize the risk of overfitting given the limited number of mortality events (n = 41), the number of covariates included in the multivariable model was restricted in accordance with accepted events-per-variable principles. HASI was entered as a continuous variable in all regression models to preserve its prognostic information.

The discriminative ability of the SI, ASI, and HASI for predicting in-hospital mortality, the primary endpoint, was assessed by receiver operating characteristic (ROC) curve analysis. The area under the curve (AUC) and 95% confidence intervals (CI) were calculated, and pairwise comparisons of AUCs were performed using DeLong’s test. All statistical analyses were performed using R software, version 4.5.0 (R Foundation for Statistical Computing, Vienna, Austria). A two-tailed *p*-value < 0.05 was considered statistically significant.

## 3. Results

### 3.1. Age-Stratified Differences in Continuous Clinical and Laboratory Variables

A total of 711 patients with STEMI who underwent primary PCI were analyzed. Of these, 457 (64.3%) were aged <65 years and 254 (35.7%) were aged ≥65 years. Baseline clinical characteristics and laboratory values are summarized in Table 1. Compared with younger patients, the elderly group exhibited significantly higher serum creatinine (1.63 ± 1.96 mg/dL vs. 1.05 ± 0.91 mg/dL, *p* < 0.001) and troponin T levels (1602.36 ± 2764.56 ng/L vs. 914.01 ± 2184.07 ng/L, *p* < 0.001), but lower hemoglobin (13.33 ± 2.20 g/dL vs. 15.30 ± 1.76 g/dL, *p* < 0.001), platelet count (214.69 ± 67.91 × 10^3^/μL vs. 243.91 ± 63.14 × 10^3^/μL, *p* < 0.001), and systolic and diastolic blood pressures (both *p* < 0.001).

In terms of physiologic indices, both the ASI and HASI were significantly higher in older patients (ASI: 45.72 ± 16.18 vs. 32.34 ± 12.01, *p* < 0.001; HASI: 0.48 ± 0.18 vs. 0.33 ± 0.13, *p* < 0.001), whereas the traditional SI did not differ between groups (*p* = 0.763). Older patients also had longer hospital stays (8.71 ± 8.30 days vs. 7.36 ± 11.94 days, *p* < 0.001) and slightly longer ICU stays (*p* = 0.001), while the duration of mechanical ventilation did not differ significantly (*p* = 0.296). No significant age-related differences were observed in CK-MB, CPK, respiratory rate, body temperature, or door-to-balloon times (*p* > 0.05).

Collectively, these findings indicate that elderly STEMI patients had greater myocardial and renal injury with reduced hemodynamic reserve and oxygenation compared with younger patients.

### 3.2. Comparison of Categorical Clinical Characteristics and Outcomes Between Age Groups

Categorical clinical characteristics and in-hospital outcomes are summarized in Table 2. Older patients (≥65 years) were significantly more likely to be female (29.9% vs. 6.6%, *p* < 0.001) and to present with higher triage severity and Killip class at admission. Specifically, elderly patients more frequently fell into Triage Class 1 (30.7% vs. 20.8%, *p* = 0.003) and Killip Class IV (33.9% vs. 17.9%, *p* < 0.001). Regarding comorbidities, the elderly group had higher rates of DM (39.4% vs. 27.4%, *p* = 0.0013), CKD (11.0% vs. 3.1%, *p* < 0.001), and CAD (12.2% vs. 7.2%, *p* = 0.0368).

In-hospital outcomes showed that the elderly had significantly higher rates of LMCA occlusion (8.7% vs. 4.4%, *p* = 0.031) and more frequent use of IABP (7.1% vs. 2.6%, *p* = 0.008) and temporary pacemaker insertion (5.1% vs. 1.1%, *p* = 0.003). Elderly patients also had higher incidences of major adverse events (26.0% vs. 10.1%, *p* < 0.001), intubation (16.9% vs. 7.4%, *p* < 0.001), CABG (2.8% vs. 0.4%, *p* = 0.012), in-hospital cardiac arrest (5.1% vs. 1.5%, *p* = 0.011), acute kidney injury (18.5% vs. 8.3%, *p* = 0.004), and in-hospital mortality (9.4% vs. 3.7%, *p* = 0.003).

These results collectively demonstrate that elderly STEMI patients presented with more severe hemodynamic compromise and experienced substantially worse short-term outcomes despite contemporary reperfusion therapy.

### 3.3. Kaplan–Meier Survival Analysis by Age Group

**Figure 1:**
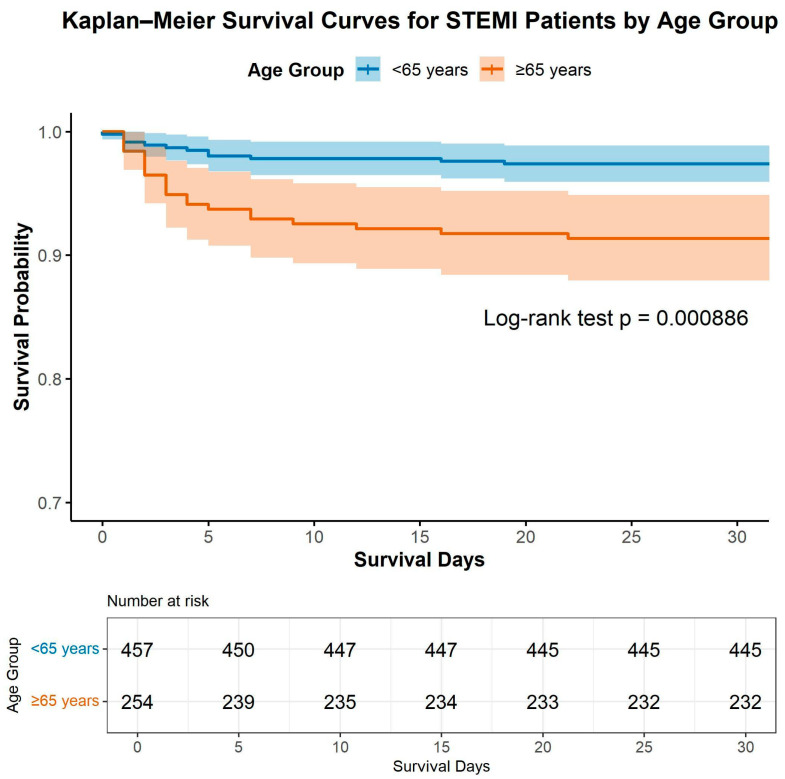
Kaplan–Meier Survival Curves for 30-Day Mortality Stratified by Age Group.

Kaplan–Meier survival analysis revealed a significantly lower 30-day survival probability among elderly STEMI patients (≥65 years) compared with their younger counterparts (<65 years) (Figure 1). The estimated 30-day survival was approximately 98% in the younger group and 93% in the older group. In this analysis, patients alive at 30 days were censored at that time point, and no patients were lost to follow-up within the 30-day observation window.

The survival curves diverged early within the first 10 days after admission and remained significantly separated throughout the follow-up period (log-rank *p* = 0.000886). This finding underscores the pronounced early mortality risk associated with advanced age in the setting of acute STEMI treated with primary PCI.

### 3.4. Multivariable Logistic Regression and Cox Proportional Hazards Analysis

In multivariable logistic regression, age ≥ 65 years was independently associated with increased 30-day mortality (OR 2.51, 95% CI 1.28–4.99, *p* = 0.0075), whereas sex showed no significant effect (OR 0.75, 95% CI 0.35–1.72, *p* = 0.471).

**Figure 2:**
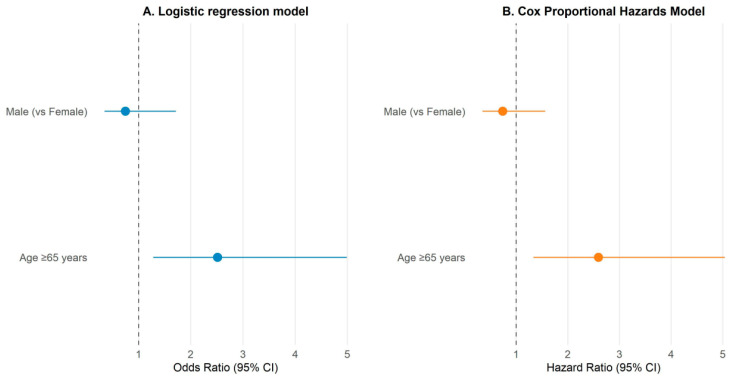
Multivariable Logistic Regression and Cox Proportional Hazards Analysis of 30-Day Mortality.

Similarly, Cox proportional hazards analysis confirmed that older age was a significant predictor of time-to-death within 30 days (HR 2.59, 95% CI 1.34–5.04, *p* = 0.0049), whereas male sex was not (HR 0.74, 95% CI 0.35–1.57, *p* = 0.431). These results affirm the strong prognostic impact of age on short-term survival among STEMI patients undergoing primary PCI (Table 3, Figure 2).

### 3.5. Univariable and Multivariable Predictors of In-Hospital Mortality

The results of the univariable logistic regression analyses for in-hospital mortality are summarized in Table S1. In univariable analysis, higher HASI values were significantly associated with increased in-hospital mortality (OR 2.35 per unit increase, 95% CI 1.62–3.41; *p* < 0.001). Other variables significantly associated with mortality included advanced age, Killip class IV, elevated serum creatinine, lower hemoglobin levels, in-hospital cardiac arrest at presentation (IHCA), longer door-to-balloon time, LMCA involvement, and multivessel coronary disease (all *p* < 0.05). Sex was not significantly associated with in-hospital mortality.

A pre-specified multivariable logistic regression model incorporating HASI and key clinical confounders is presented in Table S2. After multivariable adjustment, HASI remained independently and significantly associated with in-hospital mortality (adjusted OR 8.28, 95% CI 1.47–47.2; *p* = 0.017). Cardiogenic shock showed the strongest association with mortality (adjusted OR 8.97, 95% CI 4.00–22.2; *p* < 0.001), followed by IHCA (adjusted OR 4.14, 95% CI 1.33–12.5; *p* = 0.015).

Collectively, these findings indicate that HASI provides prognostic information beyond traditional clinical and hemodynamic risk markers and remains independently associated with in-hospital mortality after adjustment for major confounding variables.

### 3.6. ROC Analysis and Pairwise Comparison of SI, ASI, and HASI for Predicting In-Hospital Mortality

**Figure 3:**
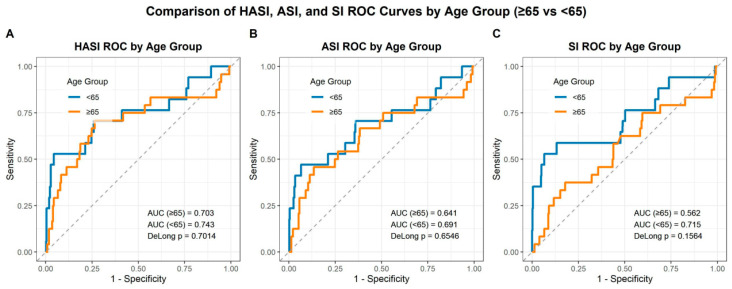
Age-group comparison of ROC curves for HASI, ASI, and SI in predicting in-hospital mortality. (**A**) HASI; (**B**) ASI; (**C**) SI. ROC curves are presented separately for patients aged ≥65 and <65 years. Corresponding AUCs and DeLong test *p* values are displayed.

ROC curve analysis evaluated the predictive performance of the SI, ASI, and HASI for in-hospital mortality (Figure 3). Across all models, HASI demonstrated the highest discriminative ability in both age groups. In patients aged <65 years, the AUC for HASI was 0.743 (95% CI 0.590–0.895), compared with 0.691 (95% CI 0.529–0.853) for ASI and 0.715 (95% CI 0.555–0.875) for SI.

Among patients aged ≥65 years, the AUC values were 0.703 (95% CI 0.566–0.839) for HASI, 0.641 (95% CI 0.501–0.782) for ASI, and 0.562 (95% CI 0.424–0.700) for SI. However, pairwise AUC comparisons using DeLong’s test revealed no statistically significant differences among the three indices in either age group (*p* > 0.05 for all). Although overall discrimination was moderate and statistical superiority was not demonstrated, the consistently higher AUC point estimates for HASI suggest a numerically favorable and potentially clinically relevant discriminative performance when applied at triage.

## 4. Discussion

In this study, we evaluated the utility of the HASI, a novel triage-based risk model, to predict mortality among geriatric patients (≥65 years) presenting with STEMI undergoing primary PCI. The principal findings are as follows: (1) older STEMI patients displayed markedly worse clinical profiles at presentation including higher creatinine and troponin T levels, lower hemoglobin, platelets and blood pressures, and higher ASI and HASI scores compared with younger counterparts; (2) age ≥ 65 years remained an independent predictor of 30-day mortality in both logistic regression (OR 2.51) and Cox proportional hazards models (HR 2.59); and (3) among hemodynamic indices, HASI yielded the highest discriminative ability for in-hospital mortality (AUC up to 0.743 in younger patients and 0.703 in older patients), albeit without statistically significant pairwise differences compared with SI or ASI. These findings warrant further commentary in the context of existing literature and practical implications.

The prognostic value of simple hemodynamic indices has been explored previously. For example, the classic SI has been shown to correlate with infarct size, microvascular obstruction and adverse outcomes in STEMI patients. Reinstadler et al. studied 791 STEMI patients and found admission SI independently predicted major adverse cardiac events at 12 months (HR 2.92) and was associated with larger infarct size on MRI [14]. Zhou et al. reported that ASI outperformed SI for in-hospital and long-term mortality among STEMI patients undergoing emergency PCI (ROC-AUC 0.805 and 0.813, respectively) [15]. More recently, the concept of incorporating hypoxia, as measured by oxygen saturation, into risk models has emerged. Hsieh et al. reported that the HASI is a strong predictor of in-hospital mortality in COVID-19 pneumonia [18,19,20].

To our knowledge, this is among the first studies to apply the HASI in a geriatric STEMI cohort treated with primary PCI, thus extending prior work in trauma, sepsis or respiratory infection settings to the acute coronary syndrome domain [18,19,20,21,22,23,24,25,26]. Our results align with these earlier studies in demonstrating that age-adjusted and physiology-integrated indices provide incremental prognostic information beyond classical vital signs alone. For instance, we found that while SI alone did not differ significantly between age groups (*p* = 0.763), ASI and HASI did (*p* < 0.001). This suggests that the physiologic burden of aging and hypoxia may amplify risk in ways not captured by HR/SBP alone. Furthermore, although pairwise AUC comparisons did not reach statistical significance, HASI consistently demonstrated numerically higher AUC values across age groups, suggesting a trend toward improved discrimination at triage. Rather than relying on marginal differences in AUC, the clinical value of HASI lies in its superior calibration, ensuring more accurate estimation of absolute mortality risk at triage. This finding is particularly relevant in the emergency setting, where reliable risk calibration is essential for early clinical decision-making, resource allocation, and escalation of care in high-risk elderly STEMI patients (Table S3, Figure S1).

The markedly worse presentation and outcomes among older STEMI patients in our cohort confirm prior epidemiologic observations: geriatric patients often have greater comorbidity burdens, reduced cardiac reserve, more frequent atypical presentation and delayed reperfusion [4,5]. In our dataset, older patients presented with greater renal impairment (higher creatinine), larger myocardial injury marker (troponin T), worse hemodynamics (lower SBP/DBP, lower SpO_2_) and required longer ICU stays and hospitalization. These features underline why advanced age remains one of the strongest prognostic factors in STEMI despite advances in reperfusion therapy.

The incorporation of hypoxia into the ASI to derive the HASI offers a pragmatic improvement in triage risk-stratification: hypoxia may reflect underlying lung disease, pulmonary congestion, low cardiac output or systemic hypoperfusion, all of which may compound the risk in STEMI [16,17]. In older patients, reduced physiological reserve means that even modest hypoxia can precipitate worse outcomes [1,2,3,4,5]. Therefore, calculating a HASI at presentation may identify high-risk elderly STEMI patients who might benefit from more rapid escalation of care, closer monitoring, or adjunctive therapies (e.g., mechanical circulatory support, advanced hemodynamic monitoring).

Moreover, the fact that age remained a strong independent predictor of mortality even after adjustment implies that beyond hemodynamics and hypoxia, age integrates multiple unmeasured risk factors: frailty, microvascular dysfunction, impaired myocardial healing, polypharmacy, and non-cardiac comorbidity [16,17]. Risk-models such as HASI may bridge the gap between simple vital-sign indices and more complex risk scores (e.g., GRACE) by offering bed-side applicability in the emergency setting.

The strengths of our study include a reasonably large single-center STEMI cohort treated with contemporary primary PCI, stratified by age group, and the novel application of HASI in this clinical context. We used routinely available triage data (HR, BP, SpO_2_, age), thereby enhancing the feasibility and potential generalizability of HASI as a rapid risk tool.

However, several limitations merit attention. First, the retrospective design introduces the risk of selection and information bias. Although we included consecutive patients and excluded those with missing key triage data, residual confounding cannot be excluded. Second, our cohort reflects a single tertiary-care center; results may differ in other systems with different triage practices, PCI timing, or patient demographics. Third, while HASI demonstrated higher AUCs, the differences between indices were not statistically significant and overall discrimination remained moderate. Therefore, HASI should not yet replace comprehensive risk models but may supplement them. Fourth, we did not externally validate HASI in this study; future prospective multicenter validation is needed. Fifth, we focused primarily on in-hospital and 30-day outcomes; longer-term prognostic value in geriatric STEMI remains to be studied.

Given the promising findings, future work should include (1) prospective validation of HASI in multi-center geriatric STEMI populations; (2) comparison of HASI with established risk scores (e.g., GRACE, TIMI) to determine incremental value in elderly subsets; (3) investigation of whether early triage interventions triggered by elevated HASI (e.g., early ICU admission, mechanical circulatory support) translate into improved outcomes; and (4) evaluation of the utility of HASI in other acute coronary syndrome subtypes (e.g., non-STEMI) or in patients managed conservatively. Additionally, future studies could explore the relationship between HASI and detailed clinical, anamnestic, laboratory, and functional variables, which may influence patient risk profiles. Identification of an evidence-based HASI cutoff to predict a higher incidence of major adverse outcomes could further enhance its clinical applicability and guide early triage decisions.

## 5. Conclusions

In conclusion, our findings demonstrate that in geriatric STEMI patients undergoing primary PCI, older age (≥65 years) portends significantly worse presentation and outcomes and remains an independent predictor of mortality. The triage-derived HASI showed numerically higher discrimination for in-hospital mortality, outperforming classic SI and ASI in magnitude, though not statistically. Given its simplicity, bed-side applicability and integration of age, hemodynamics and oxygenation, HASI may serve as a useful adjunct in early risk stratification of older STEMI patients. Further prospective validation and exploration of targeted interventions guided by HASI are warranted.

## Figures and Tables

**Table 1:** Comparison of Continuous Clinical Characteristics Between Age Groups.

Variable	<65 Years (n = 457)	≥65 Years (n = 254)	*p*-Value
Age (years)	52.25 ± 8.50 [54.00 (13.00)]	73.34 ± 6.94 [72.00 (9.00)]	<0.001 *
BMI (kg/m^2^)	26.63 ± 4.96 [26.10 (5.20)]	24.30 ± 3.49 [24.20 (4.20)]	<0.001 *
SBP (mmHg)	135.60 ± 30.04 [134.00 (37.00)]	128.09 ± 32.30 [125.00 (34.75)]	<0.001 *
DBP (mmHg)	89.06 ± 21.93 [90.00 (29.00)]	76.47 ± 20.53 [74.00 (24.00)]	<0.001 *
HR (bpm)	80.88 ± 20.00 [81.00 (25.00)]	76.32 ± 21.60 [74.00 (25.75)]	0.002 *
RR (/min)	19.49 ± 2.80 [20.00 (2.00)]	19.85 ± 2.95 [20.00 (2.00)]	0.210
BT (°C)	36.04 ± 0.66 [36.10 (0.80)]	36.05 ± 0.72 [36.10 (0.90)]	0.300
SpO_2_ (%)	97.54 ± 3.70 [98.00 (3.00)]	96.51 ± 4.00 [98.00 (3.75)]	<0.001 *
WBC (×10^3^/μL)	12.59 ± 3.84 [12.07 (4.62)]	11.11 ± 4.04 [10.38 (4.29)]	<0.001 *
HGB (g/dL)	15.30 ± 1.76 [15.50 (1.90)]	13.33 ± 2.20 [13.60 (3.00)]	<0.001 *
Platelet (×10^3^/μL)	243.91 ± 63.14 [237.00 (73.00)]	214.69 ± 67.91 [208.00 (80.00)]	<0.001 *
Creatinine (mg/dL)	1.05 ± 0.91 [0.91 (0.32)]	1.63 ± 1.96 [1.01 (0.58)]	<0.001 *
Troponin T (ng/L)	914.01 ± 2184.07 [62.60 (629.70)]	1602.36 ± 2764.56 [252.40 (1774.28)]	<0.001 *
CPK (U/L)	693.40 ± 1290.80 [199.00 (519.50)]	768.39 ± 1472.40 [206.50 (707.00)]	0.675
CK-MB (U/L)	96.46 ± 187.75 [36.00 (68.00)]	98.03 ± 149.37 [41.00 (79.00)]	0.384
SI	0.62 ± 0.21 [0.59 (0.20)]	0.62 ± 0.22 [0.60 (0.22)]	0.763
ASI	32.34 ± 12.01 [30.78 (12.28)]	45.72 ± 16.18 [43.66 (15.94)]	<0.001 *
HASI	0.33 ± 0.13 [0.32 (0.12)]	0.48 ± 0.18 [0.45 (0.17)]	<0.001 *
MV days	10.91 ± 12.49 [7 (2–14)]	8.05 ± 10.29 [3 (2–8)]	0.2959
ICU stay (days)	4.68 ± 9.39 [3.00 (1.00)]	4.82 ± 5.57 [3.00 (1.00)]	0.001 *
Hospital stay (days)	7.36 ± 11.94 [5.00 (2.00)]	8.71 ± 8.30 [6.00 (3.75)]	<0.001 *
Number of occluded vessels	2.09 ± 0.87 [2.00 (2.00)]	2.37 ± 0.89 [3.00 (1.00)]	<0.001 *
D2W (min)	74.73 ± 38.27 [69.00 (17.00)]	79.66 ± 44.02 [69.00 (16.75)]	0.170
D2B (min)	76.48 ± 38.35 [70.00 (17.00)]	81.26 ± 44.15 [70.00 (17.00)]	0.207

Data are presented as mean ± standard deviation [median (interquartile range)]. Group differences were tested using the Wilcoxon rank-sum test (Mann–Whitney U). * *p* < 0.05. Abbreviations: BMI, body mass index; SBP, systolic blood pressure; DBP, diastolic blood pressure; HR, heart rate; RR, respiratory rate; BT, body temperature; SpO_2_, peripheral oxygen saturation; WBC, white blood cell count; HGB, hemoglobin; CPK, creatine phosphokinase; CK-MB, creatine kinase-MB; SI, shock index; ASI, age shock index; HASI, hypoxia age shock index; MV, mechanical ventilation; ICU, intensive care unit; SI, shock index; ASI, age shock index; HASI, hypoxia age shock index; D2W, door-to-wire time; D2B, door-to-balloon time.

**Table 2:** Comparison of Categorical Clinical Characteristics and In-Hospital Outcomes Between Age Groups.

Variable	Category	<65 Years (n = 457)	≥65 Years (n = 254)	*p*-Value
Sex	Female	30 (6.6%)	76 (29.9%)	
	Male	427 (93.4%)	178 (70.1%)	<0.001 *
Comorbidities	DM	125 (27.4%)	100 (39.4%)	0.0013 *
	Dyslipidemia	86 (18.8%)	46 (18.1%)	0.8949
	CKD	14 (3.1%)	28 (11.0%)	<0.001 *
	HTN	164 (35.9%)	63 (24.8%)	0.0031 *
	CAD	33 (7.2%)	31 (12.2%)	0.0368 *
	Previous PCI	20 (4.4%)	11 (4.3%)	1.000
Triage Class	1 Resuscitation	95 (20.8%)	78 (30.7%)	0.003 *
	2 Emergent	265 (58.0%)	114 (44.9%)	0.001 *
	3 Urgent	96 (21.0%)	56 (22.0%)	0.746
	4 Less urgent	1 (0.2%)	6 (2.4%)	0.006 *
Killip Class	I No HF	274 (60.0%)	114 (44.9%)	<0.001 *
	II Mild HF (rales)	67 (14.7%)	28 (11.0%)	0.172
	III Severe HF	34 (7.4%)	26 (10.2%)	0.199
	IV Cardiogenic shock	82 (17.9%)	86 (33.9%)	<0.001 *
In-hospital Outcomes	LMCA occlusion	20 (4.4%)	22 (8.7%)	0.0311 *
	LAD occlusion	391 (85.6%)	221 (87.0%)	0.673
	LCX occlusion	240 (52.6%)	162 (63.8%)	0.0052 *
	RCA occlusion	304 (66.5%)	197 (77.6%)	0.0026 *
	TPM	5 (1.1%)	13 (5.1%)	0.0025 *
	IABP	12 (2.6%)	18 (7.1%)	0.0083 *
	MAE	46 (10.1%)	66 (26.0%)	<0.001 *
	ECMO	18 (3.9%)	13 (5.1%)	0.5848
	Intubation	34 (7.4%)	43 (16.9%)	<0.001 *
	CABG	2 (0.4%)	7 (2.8%)	0.0122 *
	IHCA	7 (1.5%)	13 (5.1%)	0.0113 *
	Cardiogenic shock	64 (14.0%)	68 (26.8%)	0.002 *
	Acute kidney injury	38 (8.3%)	47 (18.5%)	0.004 *
	ICU stay >5 days	45 (9.8%)	56 (22.0%)	0.001 *
	Mortality	17 (3.7%)	24 (9.4%)	0.003 *
	Readmission	17 (3.7%)	10 (3.9%)	1.000

Data are presented as number (percentage) unless otherwise indicated. Overall comparisons for Triage and Killip classes were performed using the Cochran–Armitage trend test. Pairwise comparisons for each category versus all others were conducted using Fisher’s exact test (for expected counts < 5) or Pearson’s chi-square test, with Bonferroni correction applied to adjust for multiple testing. * *p* < 0.05. Abbreviations: DM, diabetes mellitus; CKD, chronic kidney disease; HTN, hypertension; CAD, coronary artery disease; PCI, percutaneous coronary intervention; LMCA, left main coronary artery; LAD, left anterior descending artery; LCX, left circumflex artery; RCA, right coronary artery; TPM, temporary pacemaker; IABP, intra-aortic balloon pump; MAE, major adverse event; ECMO, extracorporeal membrane oxygenation; CABG, coronary artery bypass grafting; IHCA, in-hospital cardiac arrest; HF, heart failure.

**Table 3:** Multivariable Logistic Regression and Cox Proportional Hazards Analysis of 30-day Mortality in STEMI Patients.

**A. Logistic Regression Analysis**			
Variable	OR	95% CI	*p*-Value
(Intercept)	0.051	0.020–0.115	<0.001 *
Age Group ≥ 65 years	2.51	1.28–4.99	0.0075 *
Male	0.749	0.350–1.72	0.471
**B. Cox Proportional Hazards Analysis**			
Variable	HR	95% CI	*p*-Value
Age Group ≥ 65 years	2.59	1.34–5.04	0.0049 *
Male	0.739	0.349–1.57	0.430

Abbreviations: CI, confidence interval; OR, odds ratio; HR, hazard ratio;. Notes: The analyses included 30-day mortality data from 711 STEMI patients, with 41 recorded death events. Both logistic regression and Cox proportional hazards models were adjusted for age group and sex. The proportional hazards assumption for the Cox model was verified and met (*p* > 0.05). * *p* < 0.05.

## Data Availability

The data that support the findings of this study are available on request from the corresponding author. The data are not publicly available due to privacy or ethical restrictions.

## Supplementary Materials

The following supporting information can be downloaded at: https://www.mdpi.com/article/10.3390/medicina62020365/s1. Figure S1: Calibration Performance of SI, ASI, and HASI for In-Hospital Mortality Assessed by the Hosmer–Lemeshow Test; Table S1: Univariable logistic regression analysis for predicting in-hospital mortality in STEMI patients undergoing primary PCI; Table S2: Multivariable logistic regression analysis of in-hospital mortality in STEMI patients: adjusted odds ratios for HASI and clinically relevant covariates; Table S3: Calibration Performance of SI, ASI, and HASI for In-Hospital Mortality Assessed by the Hosmer–Lemeshow Test.
