# Supplementary material for: Use of the Hypoxia–Age–Shock Index at Triage to Predict Mortality in Geriatric STEMI Patients Undergoing Primary PCI
Source: Medicina (Kaunas). 2026 Feb 12;62(2):365. doi: 10.3390/medicina62020365 (PMC12943161; doi:10.3390/medicina62020365)
Supplement: Supplementary file 1 [file medicina-62-00365-s001.zip › medicina-4127861-supplementary.pdf]

Figure S1. Calibration Performance of SI, ASI, and HASI for In-Hospital Mortality Assessed by the Hosmer–Lemeshow Test.

(A)

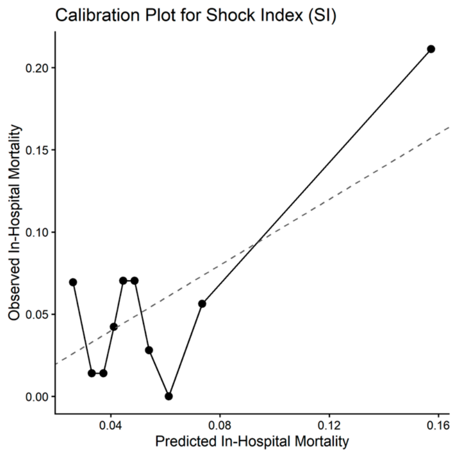

(B)

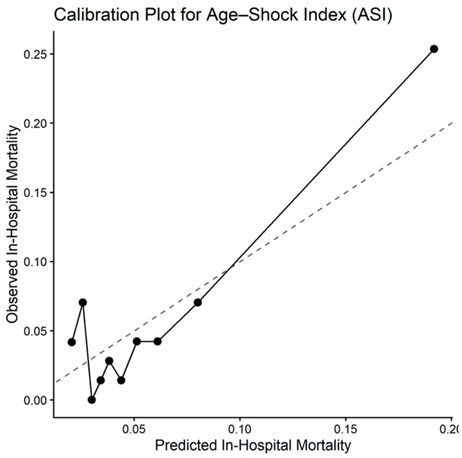

(C)

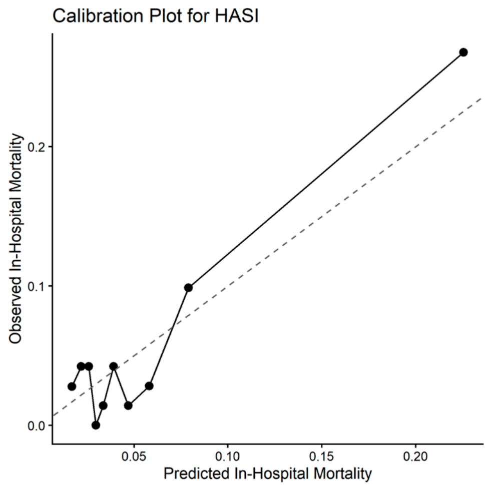

Table S1. Univariable logistic regression analysis for predicting in-hospital mortality in STEMI patients undergoing primary PCI.

Variable	term	OR	CI_lower	CI_upper	*p*_value
HASI	HASI	2.35	1.62	3.41	<0.001*
Age	Age	1.06	1.04	1.09	<0.001*
Sex	Male	0.82	0.45	1.51	0.53
KillipClass	KillipClass/IV	4.90	2.30	10.4	<0.001*
Creatinine	Creatinine	1.42	1.18	1.72	<0.001*
Hemoglobin	HGB	0.81	0.70	0.93	0.003*
IHCA	IHCA/Yes	6.20	2.80	13.7	<0.001*
D2B	D2B	1.01	1.00	1.02	0.02*
LMCA	LMCA/Yes	2.85	1.30	6.25	0.009*
Multivessel disease	Occlusion No. ≥ 2	1.90	1.02	3.55	0.041*

* $p < 0.05$

Table S2. Multivariable logistic regression analysis of in-hospital mortality in STEMI patients: adjusted odds ratios for HASI and clinically relevant covariates.

**Variable**	**Adjusted OR**	**CI_lower**	**CI_upper**	***p*_value**
HASI Score (continuous)	8.28	1.47	47.2	0.017*
Cardiogenic Shock	8.97	4.00	22.2	<0.001*
IHCA	4.14	1.33	12.5	0.015*
Hemoglobin (g/dL)	0.93	0.79	1.09	0.4
Age (years)	1.03	1.00	1.06	0.063

* $p < 0.05$

Table S3. Calibration Performance of SI, ASI, and HASI for In-Hospital Mortality Assessed by the Hosmer–Lemeshow Test.

Model	χ^2 statistic	Degrees of freedom	*p* value	Calibration interpretation
Shock Index (SI)	16.20	8	0.0396	Poor calibration
Age–Shock Index (ASI)	14.40	8	0.0720	Borderline acceptable calibration
Hypoxia–Age–Shock Index (HASI)	9.62	8	0.2926	Good calibration

Footnote: Calibration was assessed using the Hosmer–Lemeshow goodness-of-fit test. A p value > 0.05 indicates no statistically significant difference between predicted and observed event rates, reflecting good model calibration, whereas a p value < 0.05 suggests poor calibration due to systematic deviation.
